# The funhouse mirror: the I in personalised healthcare

**DOI:** 10.1186/s40504-020-00108-0

**Published:** 2021-01-05

**Authors:** Mira W. Vegter, Hub A. E. Zwart, Alain J. van Gool

**Affiliations:** 1 Institute for Science in Society, Faculty of Science, Radboud University, PO Box 6751, 6503 GG Nijmegen, The Netherlands; 2 Department of Social Sciences, Wageningen University & Research, PO Box 8130, 6700 EW Wageningen, The Netherlands; 3 Erasmus School of Philosophy, Erasmus University Rotterdam, Rotterdam, The Netherlands; 4 Translational Metabolic Laboratory, Department of Laboratory Medicine, Radboud Institute for Molecular Life Sciences, Radboud University Medical Center, Nijmegen, The Netherlands

**Keywords:** Precision medicine, Digital health, Self-tracking, Wearables, Data double, Eccentricity, iPOP, Ethics, Embodiment, Self, Personalised healthcare

## Abstract

Precision Medicine is driven by the idea that the rapidly increasing range of relatively cheap and efficient self-tracking devices make it feasible to collect multiple kinds of phenotypic data. Advocates of *N* = 1 research emphasize the countless opportunities personal data provide for optimizing individual health. At the same time, using biomarker data for lifestyle interventions has shown to entail complex challenges. In this paper, we argue that researchers in the field of precision medicine need to address the performative dimension of collecting data. We propose the fun-house mirror as a metaphor for the use of personal health data; each health data source yields a particular type of image that can be regarded as a ‘data mirror’ that is by definition specific and skewed. This requires competence on the part of individuals to adequately interpret the images thus provided.

## Introduction

*‘What lies inside all of us is more than data. It’s life …*. *the next great breakthrough will be found in each and every one of us. And what we find there will unlock mysteries, heal the sick and eradicate disease.’* This is a phrase from the youtube video published by the AllofUs research program called the ‘allofus- anthem’ and is meant to appeal to U.S. citizens to share their data on behalf of the Precision Medicine Initiative. Participation in the program is presented as an opportunity to learn about your health because you will be given access to all your health data (consent form AllofUs 2018). Scientifically speaking, research endeavours such as these build on the conviction that *N* = 1 studies are necessary and that longitudinal studies are needed based on monitoring of individuals over a period of a year or more (Schork 2015; van Gool et al. 2017). Pioneers of such large-scale *N* = 1 research, such as Leroy Hood, founding father of P4 medicine, and Michael Snyder, famous for his integrative personal omics profiling method (iPOP), emphasize the countless opportunities personal data clouds provide for optimizing individual health (Chen et al. 2012; Li et al. 2017; Price et al. 2017). Paradoxically, these data gain meaning from comparison with numerous other individuals, hence the Precision Medicine Initiative aiming to enroll one million participants.

Researchers continue to emphasize the use of wearables to collect data for biomedical research, and by doing so it has become increasingly difficult to uphold a clear distinction between biomedical research, preventive medicine and consumer technology. This raises the question what data a person should actually collect to monitor her health beyond more traditional forms of self-assessment (e.g. overall sense of wellness, weight, blood pressure, normal glass mirrors, etc.). What are the individual risk factors that require extra close monitoring? How should she make sense of this avalanche of data in every-day life and how to translate health data into concrete options for self-management and prevention? The complicating factor here is that different self-tracking devices offer specific uses of these data that allow different ‘truths’ to appear. In this paper we argue that we need to address the performative part of collecting data, which requires a responsible practice of portraying individuals in ways that allow for actual self-care. Only then we can democratize health in the way that advocates of precision medicine imagine, and live up to the true potential of personalized healthcare and the P4 medicine principles.

To illustrate the skewedness of these information sources and to highlight the inherent tensions in emerging practices, we here propose the fun-house mirror as a metaphor for the use of personal health data that may inform lifestyle choices or therapeutic interventions. We will start by providing a background narrative against which precision medicine is said to be developed, after which we will turn to the case of Micheal Snyder. His self-tracking experiment, understood from an empirical philosophical (post-phenomenological) perspective helps us to theorize what new practices Precision Medicine will give rise to and how negotiation of the data can take place.

## Actionable evidence

In 2015 Nicholas Schork discussed the necessity of one-person trials, as the ultimate way to lower the “number needed to treat” (NNT) by focusing on individual baselines. According to the author, the time was ripe for this because of three reasons: (a) the rapidly expanding -omics fields, allowing molecular profiling; (b) the increasing amount of relatively cheap and efficient devices to collect data, such as the Apple Watch™; and (c) the growing support by the government for patient engagement in medicine (Schork 2015). Since then, the Precision Medicine Initiative has been transformed into the All-of-Us research program and similar initiatives started to emerge. What began as *N* = 1 in the iPOP trial by Michael Snyder and his lab at Stanford University (Chen et al. 2012), developed into the 108 times *N* = 1 in the p100 Wellness Study by Leroy Hood (Price et al. 2017), and is currently evolving into a million times *N* = 1 in the All of Us research program led by Francis Collins (Collins and Varmus 2015). In various comments, Michael Snyder explains how the experience of being ‘wired’ with wearables and sensors is going to transform the way we manage our lives (Li et al. 2017; Snyder 2018), while Hood underscores the actionable possibilities that can be defined based on ‘personal dense dynamic data clouds’(Hood 2018; Price et al. 2017). A range of synonyms with similar meanings, such as “personalized medicine”, “precision medicine”, “systems medicine”, “P4 medicine” and “personalized healthcare”, underscore the expectations for biomarker-based health promotion and it has led to the increased uptake of self-monitoring technologies for biomedical research (Turakhia et al. 2019; Vegter 2018). Both Snyder and Hood urge us to prioritize the use of health data in promoting healthy behaviour, but the question remains whether such straightforward health benefits can really be expected for the large majority of users.

In a correspondence in *Nature Biotechnology*, Vogt et al. discuss the P100 wellness study and urge researchers in precision medicine to substantiate ‘actionable evidence’ (Vogt et al. 2018). A study published by Price et al. describes how complex -omics profiling and continuous monitoring using Fitbit™ led to lifestyle recommendations (Price et al. 2017). The authors claimed that; ‘*For each measurement in an individual that was outside the clinical reference range recommended by the clinical laboratory, the coach would recommend lifestyle changes that have been previously demonstrated to produce improvements in that marker* (Price et al. 2017, p. 753)’. Hood’s Wellness study focused on behavioural coaching to improve clinical biomarkers. In a similar vein, Snyder experimented with food and exercise in his personal omics trial to prevent his blood sugar from rising above a certain threshold when self-diagnosed as ‘prediabetic’(Chen et al. 2012). The focus on biomarkers, i.e. the reliance on bio-molecular endpoints rather than phenotypic endpoints, has been criticized by Vogt et al. who claim that this type of research leads to over-diagnosing and contributes to the “pathologization” of individuals (Vogt et al. 2018). This ties in with an important and broader observation, namely that the digitalization of healthcare in terms of big data and wearables leads many to believe there can be a quick technological fix to emerging health threats (Vegter, Landeweerd, Zwart 2020).

Not surprisingly, an entire economy is evolving dedicated to the use of smart devices for the collection of personal self-tracked data which opens new possibilities for health research. One example is the Apple Heart Study, an app-based study launched by Stanford University using Apple Watch to study over 400.000 users to identify cardiac arrhythmias (Turakhia et al. 2019). This and other initiatives show how the domain is changing in terms of volume and accessibility and how it may facilitate population screening and actionability (Chowkwanyun et al. 2018; ESC 2019; Khoury et al. 2016). The enormous amount of data that can be made available and the use of apps and smart devices for research explains why multinational companies such as Google, Apple and Amazon have rapidly moved into the healthcare domain; a phenomenon that has been referred to as the ‘googlization of health research’ (Sharon 2016).

Against this backdrop we want to take a closer look at the recent work of Michael Snyder because his position represents a unique situation, allowing him to operate at the intersection of scientific research and real-world self-experimentation while at the same time becoming an advocate for precision medicine. Snyder’s personal narrative offers a unique window into a potentially technology-driven future. We focus on his online testimonies because we believe they reveal the extent to which such practices are performative. Our analysis focusses on practices of the self in the era of digitalization, and may be regarded as an online ethnography with *N* = 1, or N = me (Hine 2008). Snyder’s story does not serve as evidence for the success of precision medicine, but rather as a case-study to conceptualize the meaning-making process that is presupposed in these technologies. First we summarize the story of Michael Snyder and his self-tracking experience, after which we conceptualize his experience in light of these practices of the self.

## N = me

Technically speaking, self-tracking has found its paragon in Stanford University’s genetics department. In 2012, Chen et al. published a paper on integrative personal omics profiling (iPOP) following a single individual over a 14-month period ‘*to interpret healthy and diseased states by connecting genomic information with additional dynamic omics activity’* (Chen et al. 2012, p. 1293). This individual turned out to be Prof. Michael Snyder, the director of the Department of Genetics (Snyder 2012). The iPOP research turned out to be the ultimate self-monitoring event. Four publications from the Snyderlab describe a detailed story about Michael Snyder as a subject of scientific research, flanked by an impressive series of Snyder’s public and academic lectures that foreground his personal narrative. Snyder’s self-tracking story starts with the famous Chen et al. paper on the omics-profiling method (n = Snyder), followed by a paper on digital health and wearables (N = Snyder + 46), a paper on tracking ‘glucotypes’ (N = Snyder + 56), and a more recent paper on the ‘exposome’ (N = Snyder + 14) which is about measuring everything there is to know about environmental exposures (Chen et al. 2011; Hall et al. 2018; Jiang et al. 2016; Li et al. 2017). At the time, the iPOP paper was so extraordinary because of Snyder’s ability to self-manage, thanks to the iPOP method; sequencing his genome and continuous monitoring enabled him to prevent the development of diabetes, something he feels was unexpected because he was unaware he was at risk (Snyder 2012). His personal success with self-monitoring data led him to develop a research program involving wearables and other items belonging to the ‘internet of things’.

Michael Snyder and his extensive self-monitoring research shows two processes of “meaning-making” that take place in parallel realities. Data are validated by biomedical research, but they are also shaped and influenced by the lived, phenomenological self. Snyder negotiates exposures (the exposome), physiology (heart rate), diets (glucotypes) and other scientific methodologies materialized by these wearables, in constant comparison with the very mundane reality of everyday life. In an early interview, Snyder narrates how his mother took part in the iPOP project and how her genome led him not to worry too much about carrying similar mutations, since she had made it to 83 years old at the time of the interview (Snyder 2012). It also tells the story of how the iPOP trial led him to look into obtaining supplemental life insurance. In more recent public testimonies, when Snyder talks about his extensive research, we witness him trying to make sense of the research data in the context of his own life and family history, addressing genetic predispositions in phrases such as ‘*no one in my family was ever overweight, however on diabetes I’m on the far end of the spectrum*’ Big Data and Health - Keynote by Michael Snyder - YouTube 2018). By adding wearables to the research – wearing over nine tracking devices while travelling extensively (as a prominent academic) – it is also a story about the bodily impacts of global mobility, for instance when he explains how his blood oxygen responds to air travel. And when at one point his blood oxygen level dropped more than usual on a flight to Oslo and did not return to baseline after landing, he ‘*knew something was up*’ Deep Omics Profiler, Mike Snyder, Now Turns to Wearables | Mendelspod 2017). He could pinpoint the cause of these deviations, based on numerous data, to an incident where he was working outside with his brother in the summer of 2015, putting up fences, when he was probably bitten by a tick, causing Lyme disease. A low-grade fever in combination with heightened heartrate induced him to seek treatment. Although the reader might be aware of the complexities that come with diagnosing Lyme disease, to Snyder this serves as a testimony that we all need to live by these baselines in order to safeguard our health.

Furthermore, Snyder has identified himself as being ‘pre-diabetic’ since the first iPOP trial: this resulted in his third publication, on using continuous glucose monitoring together with a nutrient challenge to identify glucose misregulation. Regarding this trial, Snyder explains that when he stopped running, his blood sugar kept ‘*creeping up ever since*’, and when he tried weightlifting, which is considered beneficial, it did not work for him personally Big Data and Health - Keynote by Michael Snyder - YouTube 2018). Thus these daily routines are informed by the data, yet at the same time they seem to concern mundane worries about healthy lifestyles. Thus, the ‘Snyderome’, a catchphrase used to refer to the end product of Michael Snyder’s genomic journey, is just as much a self-tracking as it is self-care (Snyder 2012; Zwart 2018).

To what extent, then, can Snyder’s case really be regarded as an exploratory pilot for things to come? For instance: Snyder is obviously well-versed in data interpretation, and he holds direct access to the measured data. In contrast to the Snyder-case, Estrin & Juels show how difficult or even impossible it is for most extra-mural individuals (for all those citizens who are not Stanford professors) to retrieve data from their devices without it already being framed in a certain way. Rather than direct measurements of skin temperature or heart activity, the user first has to upload the information for analyses, and it is often preformatted and exploited by the makers of the app (Estrin and Juels 2016, p. 45). Each of these lifestyle-apps is concerned with certain parameters, for which data are collected to form a particular (and often skewed image) of our health or lifestyle. Haggerty and Ericson (2000) argue that these data doubles are subject to a form of pragmatics; they are defined according to how useful they are for discriminating among populations. The display in your watch shows you what the producer or institutional context deems necessary for you to see in order to promote healthy behaviour, and what data they consider necessary to gather for their research. Your data is tailored to ‘their’ system, ultimately subjecting self-experimentation to their research framework.

The Quantified Self movement, an international movement of individuals who aim to ‘live by numbers’, is known for its ‘soft’ resistance against the frameworks offered through self-tracking devices (Nafus and Sherman 2014; Sharon and Zandbergen 2016). Nevertheless, as suggested by Lucivero and Prainsack, when self-tracking data enter the consumer market, a variety of social factors make the collected data and suggested actions medically relevant (Lucivero and Prainsack 2015, 48):

*‘A genome scan reveals information that is medical, genealogical and recreational. And those who scan and interpret the data are not distinct bodies of experts, but instead, novel configurations of geneticists, customers, ethicists, bioinformatics experts and new media executives* (Lucivero and Prainsack 2015, p. 47)*.*’

Even for an individual like Michael Snyder, the only way to make sense of his self-tracking results is by consulting data provided by numerous others, and by confronting norms set by other experts. Thus we see that self-experimentation, even when conducted at Stanford, is never merely scientific, but also involves a social and normative dimension (comparative self-assessment).

## Tracking the self

**Fig. 1:**
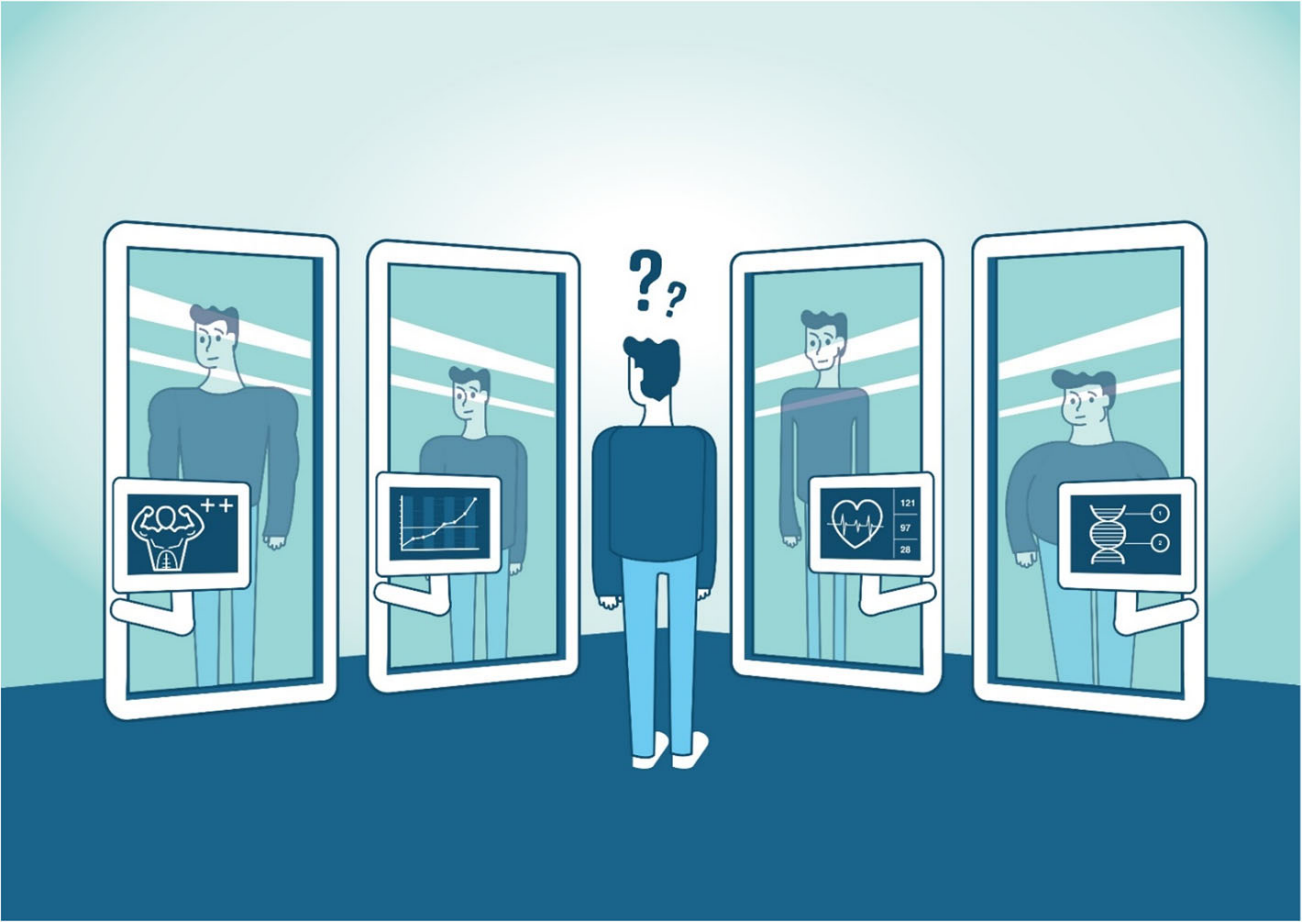
Data mirrors provided through personalised healthcare. The funhouse mirror: The I in personalised healthcare. Illustration by LizaRenee https://www.lizarenee.nl/

Building on the work of Michel Foucault and others, we see self-tracking in terms of practices of the Self, focussing on how subjectivity is constituted with the help of novel techniques (self-tracking devices), paying special attention to the normative dimension; how individuals use these techniques to constitute themselves as responsible subjects via self-examination and self-management. We argue that high precision tracking devices offer a kind of digital mirror for self-assessment. Humans have developed and employed various types of mirrors, starting with metal or glass mirrors in the literal sense, and precision medicine claims to offer more sophisticated devices, allowing us to see what is beneath our surface. Yet, we argue that precision medicine actually offers multiple mirrors, while each mirrors entails biases and refractions, so that we actually end up with a series of digital mirrors: something like a funhouse mirror or mirror maze, which implies that interpretative skills are required to come to terms with the multiple mirrors we are facing. The question inevitably emerges whether self-tracking devices result in empowerment or subjectivation, but from a Foucauldian perspective, the answer is: both. The production of a Self becomes a multi-faceted negotiation, a contest between empowering and disempowering aspects of technologies (Fig. 1). Speaking in terms of a mirror metaphor is meant as a heuristic and conceptualizing move; this paper attempts to add to our understanding of precision medicine by introducing the funhouse mirror as a discourse metaphor which specifically aims to make its underlying principles the object of criticism and debate (Gibbs and Cameron 2007; Steen 2013).

Self-monitoring gives rise to a ‘data double’, a digital copy of *N* = 1 (Haggerty and Ericson 2000). While it may seem as if this copy is singular it is made possible against a backdrop of many other individuals sharing their data as well. In the context of (self-)monitoring practices and endeavors such as the P100 study, a whole range of choices are made by the providers of the self-tracking devices, also concerning the ways in which the data are collected. These choices include setting the clinical reference range and deciding what lifestyle changes should be recommended based on genetic predispositions. Even though in the case of the P100 trial the fitbit allows multiple uses (multistability), it has a disposition to be used in a certain way and to assemble the data double in a way the researchers deem fit – the idea of technological intentionality (Ihde 1993, p. 54; Verbeek 2001, p. 136). As Verbeek explains, intentionality means that technologies provide a framework for human actions and have a certain influence on those actions: ‘*This influence does not have the character of a determinism but rather that of an inclination or “trajectory”. Technologies want people to do things in particular ways, as it were: they have a certain “intention” and promote this intention among their users*.’ (Verbeek 2001, p. 136) Technologies are designed for specific uses, as Van den Eede explains: ‘*Glasses, for example, enhance eyesight but reduce, for one, motility and flexibility when doing sports.’* (Van Den Eede 2015). It is pointed out that design for one feature (enhancing eyesight) may reduce that of another (doing sports); this is why we argue that data doubles offer a skewed image of the self, foregrounding certain aspects of the self at the expense of others.

In Snyder’s self-experiment, the research framework and the drive towards self-monitoring seem perfectly aligned in the technology; this is exceptional, as Nafus and Sherman point out when studying the Quantified Self movement – personal goals usually do not align with what the device allows for which elicits the discovery of where the frame falls short ‘*the frame breaks’* (Nafus and Sherman 2014, p. 1789). Holding on too tightly to a specific framework – for example, measuring weight – can frustrate what the individual is trying to accomplish. When it triggers feelings of unworthiness, for instance, it may lead the user to stop using the device. On the other hand, the same ‘frame’ may lead to different uses, such as calorie-counting apps that are used to sustain anorexia (Ruckenstein 2014; Van Den Eede 2014). Either way, the data double framed by the device may facilitate very particular self-practices, depending both on the design of the technology and the user.

The intimate relationship Snyder holds with his data doubles can be understood through the work of Deborah Lupton on self-tracking cultures. According to this author, the self- tracking process is cyclical and the data double plays a crucial role:

*Data doubles are also recursive and reflexive. Self- trackers reflect upon their data and seek to make sense of them. A feedback loop is established, in which personal data are produced from digital technologies which then are used by the individual to assess her or his activities and behaviour and modify them accordingly*. *Data doubles, therefore, are both constituted by the body and self and in turn serve to re- constitute the body and self.* (Lupton 2014, p. 82)

This re-constitution, as described by Lupton, is a process similar to being confronted with a mirror. Phenomenologically speaking, these individuals ‘discover’ themselves through the tracking devices, a process which is explained by Van den Eede as follows:

*In tracking one’s physical exercise patterns, for instance, a data set about distance, location, speed, calorie burning, etcetera is constituted. In this constellation of data, then, the exercising subject may find oneself back. It is as if the tracking is there first, and only in a second instance the “self” comes about.* (Van Den Eede 2015, p. 145)

Similar to the Snyder example, the story emerges retrospectively, in hindsight. Although Van Den Eede emphasizes that Self-experience is always prior to the data double, the data double does allow us to know ourselves in new ways:

*Even before one observes any data, the “world” and the “I” have already been found throughout, for example, the running activity. But still, the added “data double”* (Haggerty and Ericson 2000; Ruckenstein 2014)*) that the tracking brings about, may be seen to follow a similar pattern. One discovers oneself through the self-tracking technology—because one always discovers oneself reflexively*’. (Van Den Eede 2015, p. 145)

Self-monitoring constitutes a Self. For instance: in the interaction with the data double, Snyder is reconstituted as a tracking subject. Data doubles re-constitute the self in a certain way, usually not the way the subject perceived herself or himself prior to the technology. The data double is a new kind of mirror, a data mirror, albeit a skewed one.

## Eccentricity

As indicated, precision medicine offers a digital mirror as a medium for self-reflection. The mirror experience offered by precision medicine can be considered as a new chapter in a long history of mirror experiences involving devices developed to foster self-reflection and therefore of key importance of fostering practices of the self. The mirror experience is intimately connected with what has been thematised the ‘eccentricity’ of human beings (Plessner 1928). We do not coincide with ourselves, but actively relate to ourselves, work on ourselves and reflect on ourselves. The mirror experience allows us to view ourselves from an external position, as it were: we have the ability to self-assess, and to look at ourselves from a certain distance and in a critical manner. Via mirrors, we can work on ourselves, criticize ourselves or, in the case of narcissism, fall in love with ourselves, up to the point of becoming completely self-obsessed and self-absorbed. Michel Foucault emphasised, however, that eccentricity is not an anthropological constant, but a historical “variable”. He describes how, in various political and historical settings, individual use various subjectivation devices to constitute themselves as responsible subjects. Specular devices may give rise to “techniques of the self” and may function as a tool for looking critically at ourselves, caring for ourselves and working on ourselves (Foucault 1984). Thus, human eccentricity co-evolves with technological developments. Mirrors have played an important role in the shaping of human eccentricity and the emergence of self-reflection and self-consciousness. Mirrors as instruments for self-objectification literally enable us to take a critical look, turning the me (‘*moi*’) into an object of criticism and debate. Our mirror-image is our first double: our mirror-double.

One problem with mirrors is that we can only see part of ourselves. A mirror image is a ‘partial object’. Over the course of history, mirrors have become increasingly sophisticated, presenting an increasingly detailed portrait of ourselves, but there will always be a ‘reverse’ or obfuscated side. Moreover, the specular experience is never a completely dual relationship. The mirror image allows us (or even forces us) to compare ourselves with others, for instance with the images of superbly healthy and beautiful men and women presented to us by billboards and commercials. Furthermore, mirrors may show us something that we do not know about ourselves, something beyond the surface of our face or silhouette (our gestalt). The basic conviction of physiognomy (also known as anthroposcopy, i.e. face-science, a nineteenth-century research field) is that the face provides a window into our hidden Self. Our face (in the mirror) reflects our inner condition, as it were. The mirror is a tool of daily self-diagnostics.

Mirrors have evolved dramatically over time, from the polished metal mirrors of ancient eras to the glass-silvered mirrors of the nineteenth century. Modern mirrors reflect an extremely detailed, high resolution portrait of ourselves and are mass-produced, so that the use of mirrors as a contrivance in practices of the Self has been transformed from a more or less elite practice into mass culture. The mirror, notably the glass-silvered mirror, has thus become a universal contrivance exemplifying human eccentricity and fostering practices of self-reflection. Optical mirrors allow us to see ourselves from an outsider’s point of view. They produce a more or less ‘objective’ portrait of ourselves, for in our mirror image we (our bodily selves) actually become our own object. Mirrors may proliferate and multiply, moreover, giving rise to the experience of being surrounded by critical mirrors, urging us to take a critical look at ourselves and to improve our way of living.

Around 1900, a new type of mirror, namely psychic mirrors, emerged in the form of personality tests, such as the Myers-Briggs personality test, based on Carl Gustav Jung’s theory of personality types (Jung 1921). Such tests are mirrors which confront us with something we do not know about ourselves: our personality (in terms of extravert or introvert for instance); our alter ego; our ‘Mr. Hyde’; that which is not reflected by optical mirrors (our personal hidden Vampire as it were). Psychic mirrors have their blind sports as well: some aspects of ourselves will be obfuscated rather than revealed by test results. Again, this is not a purely dual relationship as my personal test results will be compared with standards of normalcy. It is only by comparing my test results with those of others that they can be used for self-reflection and self-care.

A new type of mirror is evolving currently: the mirror provided by self-tracking practices. This again builds on the experience of human eccentricity, while emphasising that eccentricity is not a given, but shaped and enacted as a by-product of the technologies we develop and use. Self-tracking mirrors are digital mirrors, producing a data double. Once again, eccentricity is both an outcome and a condition for technological developments. We develop digital self-tracking mirrors because of the urge to reflect upon ourselves, to care for ourselves and work on ourselves, and the technologies we subsequently produce allow us to do so, but in a particular manner. New technologies may produce portrayals that are increasingly detailed and precise, but each portrayal will have blind spots. Our world of mirrors is like a Möbius-ring, for there is always a reverse side. An optical mirror only allows us to see a part of ourselves (our face, our façade), but in the case of data doubles in self-tracking practices, the blind spots may prove more difficult to discern. We will never be completely transparent to ourselves. This is not a purely dual relationship, moreover, because self-tracking only makes sense when it allows for comparison with others (other self-trackers or the generalised community of self-trackers).

This ‘third’ position, this ‘other’ of self-reflective practices, enabled by optical, psychic and digital mirrors, may even become an obsession – a famous motif (the doppelgänger) in stories, novels and movies. We may have the impression that the mirror image is following us, to the point of haunting or stalking us. Rather than being the ones who see, we have the impression of constantly *being seen*, because mirrors tend to multiply. The sudden confrontation with the mirror image (my mirror self, my specular double) may be an uncanny experience, and this applies not only to optical mirrors (Freud 1919), but to psychic and digital mirrors as well. Instead of being the one who sees, I am *being seen*. Instead of being the one who cares and reflects, I am *being monitored and surveyed.*

## Flexible embodiment

The eccentricity shaped by the mirror points to an important observation: the data double shapes and enacts how *I* relate to *myself*. Each data double, each mirror image, encompasses a specific idea of what the body is (ontology) in terms of genes or biomarkers etc.; and how it can be managed and cultivated (ethics as practice of the Self). Therefore, in order to address the normative challenges involved, we also have to understand how quantification affects experiences of embodiment (the relationship between body and Self). Self-tracking allows for a multiplicity of selves to be reflected. Consider the diversity of approaches for self-tracking and the multitude of data doubles that can act as a mirror: a mirror maze emerges (Lupton 2014, p. 83). One way of theorizing the self in the context of the digital is through ‘reverse embodiment’(Shah 2012, p. 206; Turkle 1997). Shah has analyzed the vicissitudes of the techno-social subject in the context of cyberculture, as well as subjectivity in different digital contexts. The author describes the process of interacting with an online double or avatar as follows:

*This is a process of reverse embodiment where the presumed original is now re-shaped and re-configured to suit the imaginations and narratives of the avatar. Such a phenomenon is perhaps possible only in the domains of the cyberspace. Also, the cyborg, generally presumed as residing in the physical body, is now relocated in this two-way process, at the borders where it not only facilitates meaning but also realises itself in the process of facilitation. While the metaphor of the flow has often been used to try and describe this relationship, a network perhaps is a better way of understanding this transactional relationship. The avatar becomes a set of digital attributes – structured as well as unstructured; scripted as well as non-scripted – that can now each travel through different trajectories of personal extension and inter-personal interaction. Different processes, desires or interests of the self draw distributed representations, each mapping back upon the biological body to change and reshape the practices of the body.*

Shah describes this mapping of the online (mirrored) bodies onto physical bodies as a process where the Self adapts its self-image and behaviour in accordance with an emerging online narrative. That relationship between self and mirror-image should not be framed in terms of original and representation. Shah addresses reverse embodiment in the context of multiplicity, notably the idea that there are different selves or different subjectivities constituted in the interaction with the online world. Whether it is about games or social platforms, Shah argues that competing subjectivities or notions of the self are constantly being reconfigured.^1^

An understanding of this mapping unto individual bodies, of how self-tracking relates to self-experimentation, demands a conceptualization of embodiment. Consider how self-tracking can connect with an embodied experience, e.g. running with a heartrate monitor while experiencing exhaustion; the monitor can help to sustain the running activity by finding the ‘right’ heartrate and inform the running practice. Lupton describes that in such a process, neither the information from the device nor the senses are taken as authoritative. Rather, meaning is negotiated on the basis of two complementary sources of information (Lupton 2016, p. 5). This negotiation challenges the presumed split, where an ‘I’ can objectify his or her own body. Such a split suggests a distinction between the body as *object* and the body as *experienced* – which is shown to be problematic by Annemarie Mol (Mol and Law 2004). Self-monitoring incites us to look at ourselves as ‘other’, as an objectified version upon which we may wish to act. While at the same time we cannot disconnect from the body as (un-) consciously experienced. Van den Eede explains our perception as follows in his chapter ‘Tracing the Tracker’; ‘*We perceive in an embodied manner an however objectified version of our embodiment*’. (Van Den Eede 2015, p. 151) In the case of the running activity, the abstract body (heartrate) is mapped back unto the experiential body (‘I’m running too fast’).

Different kinds of abstractions can be mapped back unto the body. Mol and Law explain that medicine does not offer a unifying theory concerning isolated bodies. Rather, medicine entails a variety of diagnostic and therapeutic interventions into lived bodies (Mol and Law 2004, p. 58). Each branch of biomedicine seeks to know the body, but in different ways; by directing its gaze - the pathological, genetic, physiological or metabolic gaze - the gaze involves both observation and manipulation (Mol 2015, p. 68). And thus biomedicine enacts, through its discourse, its gaze, its materials and methods, different ideas of what a body is (Mol 2002). Mol argues that the body both acts and is being enacted, and is therefore a complex configuration of substance and activity. Her ethnographic research on diabetes further clarifies this concept: in the context of the question about what the body is made to be when it is constantly responding and acting to prevent hypoglycaemia (dangerously low blood sugar levels), she meticulously explains how patients with diabetes maintain a constant blood sugar level through an intimate relationship with the glucose-measuring device and by counteracting the threat of hypoglycaemia in their daily routines. What is interesting is that the instrument, the glucose monitoring device, gives rise to a form of self-awareness on which Mol reflects as follows:

*But from the ethnographer’s point of view the most interesting relation between objectivity and subjectivity comes with the use of measurement machines to train inner sensitivity. In training programmes people are told to guess their blood sugar levels first, before they measure them. The object is not to turn them into accurate number-guessers, but to encourage them to stop whatever they are doing in order to feel their bodies from inside. It is to seduce them into practicing self-awareness.* (Mol and Law 2004, p. 47)

The instrument facilitates the individuals’ relationship to themselves. This requires quite some effort on the part of the individual involved. As Mol argues: ‘Machines only become instruments if the body can manipulate them and incorporate them in its actions’ (Mol and Law 2004, p. 51). Given the different interpretations of what a body is and the multitude of instruments that make these interpretations actionable, it becomes clear that in practice the body can be part of multiple realities. Self-tracking devices inherently prioritize one reality over others. In the case of Michael Snyder, each wearable and each self-tracking device in practice enacts a specific form of embodiment. Snyder is a breathing body (the exposome), a body that travels (heartrate) and a body that struggles with different diets (glucotypes). And each of these bodies is enacted through one of the devices used for the iPOP trials.

It has become clear that wearables are unable to construct a personal narrative in and by themselves, nor do they configure a single self, or enact just one body. The process of fitting the numbers back into the individual’s daily lives and practices is what provides context and meaning (Sharon and Zandbergen 2016). Fitting numbers back onto individual bodies, we argue, is what constructs different subjectivities. The funhouse metaphor emphasizes the intrusiveness that accompanies the implied distinction: the object-body fed back to the lived-body, because in this process the ‘framing’ or distortion takes place while the data double continues to be constitutive for a self and the relationship to the personal self. Not only does the tracker experience her/himself expressed in data, he or she also finds her/himself defined by others. The funhouse mirror metaphor underscores that the self-monitoring practice disproportionately emphasizes very specific practices of self while devaluing others. Self-perception can be informed by many and at times competing data doubles, each of them grounded in a different ontology. From Snyder’s self-tracking experiment, we learn how he reclaims and reinterprets the data in terms of his own experienced reality, at times focusing on the data (the laboratory world), while at other times fore-fronting his personal experience (the phenomenological world). He is both researcher and participant, both the ‘I’ and the ‘me’. He is, in short, an eccentric researcher.

## Conclusion

We began our investigation by asking how to translate health data into concrete options for self-management and prevention? Our response has been that, besides possible positive impacts on health, these technologies first of all produce a series of mirror images, affecting the way we see ourselves. Self-tracking devices play a performative role, giving rise to new practices of the Self. Wearables do not construct a personal narrative in and of themselves, for this requires active interpretation by the subjects involved, nor do they configure a single self or a particular body because, as indicated, these practices give rise to multiple mirror-images. The process of fitting the mirrored and quantified Self back into our lifeworld requires negotiation. Our interactions with our tracking devices can best be compared to the confrontation with a funhouse mirror, or rather: a series of funhouse mirrors. The ‘funhouse mirror’ is a metaphor which enables an ethical analysis of the tailoring of self-tracking data, notably by indicating how particular devices may entail a prioritisation if one particular reality over others. As a mirror, a self-tracking device fosters an active relationship to ourselves – which we elaborated as *eccentricity* – but it may also disproportionately reflect particular aspects of the body at the expense of others. The extensive self-monitoring practice developed by Michael Snyder points to a tension between two processes of meaning-making, as data are validated by biomedicine but also shaped and influenced by the lived, phenomenological self. The eccentricity of self-tracking mirrors implies that these devices encourage me to critically assess *myself*. Each data double, each mirror image, entails a specific idea of what the body is (ontology) and how it can be managed and cultivated (practice of the Self). Snyder is a breathing body (the exposome), a body that travels (heartrate) and a body that struggles with different diets (glucotypes). The scientific methodologies materialized by these wearables each account for a particular mode of being (a particular ontology), and they must be negotiated constantly in order to maintain a coherent sense of embodiment.

When scientific endeavors such as the p100 project use genomics research markers to accommodate ‘actionable possibilities’, we need to ask ourselves whether Precision Medicine should allow self-tracking to become such a powerful medium for collecting health data. Our answer would be that the performativity of such data (in terms of actionable possibilities) in personal lives should be made more transparent. As we propose in this paper, emerging self-tracking technologies enact many different and at times competing ontologies upon the body, each of them giving rise to a different experience of Self. Making the data fit into our lives therefore requires interpretative skills that must be fostered and facilitated in order to prepare ourselves for future forms of healthy citizenship.

## Data Availability

Not applicable.

## Abbreviations

iPOP: integrative personal omics profiling
NNT: number needed to treat

## Footnotes

1 Shah draws upon Sherry Turkle’s work. Turkle addressed the possible benefit of the idea of multiplicity using Daniel Dennett’s idea of a flexible self and his work on ‘multiple drafts’ theory of consciousness. Turkle stated: ‘*As when several versions of a document are open on a computer screen, a user is able to move among them at will. Knowledge of these drafts encourages a respect for the many different versions and imposes a certain distance from them*.’ (Turkle 1997, p. 1105) Turkle convincingly argued in the late 1990s that computational environments allow cycling through different aspects of self and that they have become a way of life in which parallel definitions, like thinking about one’s identity in terms of parallel lives, becomes a habit of mind. (Turkle 1997, p. 1109) Turkle additionally argued, along the lines of Haraway (Haraway 1991), that split selves make open communication possible among various aspects of the self, in order to encourage *‘an attitude of respect for the many within us and the many within others’.* The downside, however, is that the data double and the definition of the subject in self-tracking are created by the few – the scientific establishment – and are mapped back unto individual bodies, which is often classifies as Biopower. (Foucault 1976) Multiplicity in the context of precision medicine would help individuals to better understand the meaning of their data, and to *‘move among*’ the data doubles, as Turkle suggested.
